# Comprehensive elaboration of glycemic variability in diabetic macrovascular and microvascular complications

**DOI:** 10.1186/s12933-020-01200-7

**Published:** 2021-01-07

**Authors:** Bao Sun, Zhiying Luo, Jiecan Zhou

**Affiliations:** 1 Department of Pharmacy, The Second Xiangya Hospital, Central South University, No. 139 Middle Renmin Road, Changsha, 410011 Hunan China; 2 Institute of Clinical Pharmacy, Central South University, Changsha, 410011 China; 3 Institute of Clinical Medicine, The First Affiliated Hospital, University of South China, No. 69, Chuanshan Road, Hengyang, 421001 Hunan China

**Keywords:** Glycemic variability, Diabetes mellitus, Diabetic macrovascular and microvascular complications, Therapeutic strategies

## Abstract

Diabetes mellitus is the major risk factor for the development of macrovascular and microvascular complications. It is increasingly recognized that glycemic variability (GV), referring to oscillations in blood glucose levels and representing either short-term or long-term GV, is involved in the pathogenesis of diabetic complications and has emerged as a possible independent risk factor for them. In this review, we summarize the metrics and measurement of GV in clinical practice, as well as comprehensively elaborate the role and related mechanisms of GV in diabetic macrovascular and microvascular complications, aiming to provide the mechanism-based therapeutic strategies for clinicians to manage diabetes mellitus.

## Introduction

Diabetes mellitus characterized by hyperglycemia is a major chronic metabolic disorder primarily caused by defects in insulin secretion, insulin action or both [1]. Globally, it is estimated that 463 million people have diabetes and this number is projected to reach 700 million by 2045 [2]. These individuals have at least a twofold increased risk of cardiovascular events compared with those without diabetes mellitus, playing a leading role in diabetes-related morbidity and mortality [3–5]. Moreover, diabetes mellitus contributes to the development of macrovascular complications, such as coronary artery disease, peripheral vascular disease and cerebrovascular disease, and microvascular complications, including retinopathy, nephropathy and neuropathy [6].

Although HbA1c remains the gold-standard assay for assessing glycemic control, it is not a complete expression of glycemic status [7]. Glycemic variability (GV), representing an integral component of glucose homoeostasis, is emerging as an important metric to assess glycemic control in clinical practice and without doubt now being recognized [8]. Recent epidemiological evidence suggested that GV was associated with higher risk for cardiovascular events among individual with diabetes mellitus, and the biological plausibility of the association between GV and the progression of diabetic vascular complications had been proposed [9–12]. However, the importance of GV on diabetic complications is still under debate due to inconclusive evidence [13, 14].

Our previous study also indicated that GV was associated with cardiovascular events and hypoglycemia [15, 16]. Although it has not yet been clearly identified as an independent risk factor for diabetic complications, the role of GV in diabetic complications has attracted a lot of attention. In this review, we summarize the main categories and measurement of GV in clinical practice, as well as comprehensively elaborate the role and related mechanisms of GV in diabetic macrovascular and microvascular complications, aiming to provide the mechanism-based therapeutic strategies for clinicians to manage diabetes mellitus.

## Metrics and measurement of GV

Generally, GV is defined by the measurement of fluctuations of glucose or other related parameters of glucose homoeostasis within a given time interval. However, currently, there is no consensus on the optimum method to characterize GV [17]. Although various metrics quantifying GV have been introduced, many of them are not well understood [14, 18]. Therefore, metrics that effectively describes GV will be desirable. There are mainly two categories of metrics: long-term GV, based on serial determinations over a longer period of time, usually involving HbA1c, serial fasting plasma glucose (FPG) and postprandial glucose (PPG) measurements, and short-term GV, assessed by both within-day and between-day GV (Table 1).

**Table 1:** The metrics and measure of GV

Various metrics and measure of GV	Description or definition	References
Long-term GV		
CV	Variation around the mean blood glucose of HbA1c, FPG and PPG between sequential visits	[19]
SD	Magnitude of variability relative to mean blood glucose of HbA1c, FPG and PPG between sequential visits	[19]
VIM	Based on logarithmic curve fitting (the natural logarithm of SD over the natural logarithm of the mean)	[22]
Short-term GV		
Within-day or between-day GV		
CV	Variation around the mean blood glucose	[18]
SD	Magnitude of variability relative to mean blood glucose	[18]
LBGI/HBGI	Measure of frequency and magnitude of hypoglycemia or hyperglycemia	[31]
ADRR	Sum of the daily peak risks for hypoglycemia and hyperglycemia	[32]
Within-day GV		
MAGE	Mean differences from peaks to nadirs	[23]
MAG	Absolute differences between sequential readings divided by the time	[8]
CONGA	Difference between a current blood glucose reading and a reading taken hours earlier	[25]
TIR	Percentage of time spent within the target glucose range of 3.9–10.0 mmol/L during a 24-h period	[26, 27]
Between-day GV		
MODD	Absolute differences between two glucose values measured at the same time with a 24 h interval	[28]
AGP/IQRs	Distribution of glucose data at a given timepoint	[29, 30]
Measuring method of GV		
SMBG	Reflected blood glucose fluctuations on the timescale of hours or days	[20]
CGM	Interstitial glucose measurements at 5 min intervals	[20, 34]
Flash glucose monitoring	Measured interstitial glucose and indicated direction and speed of glucose change	[36]

*GV* glycemic variability, *CV* coefficient of variation, *SD* standard deviation, *FPG* fasting plasma glucose, *PPG* postprandial glucose, *VIM* variation independent of the mean, *LBGI* low blood glucose index, *HBGI* high blood glucose index, *ADRR* average daily risk range, *MAGE* mean amplitude of glycemic excursions, *MAG* mean absolute glucose, *CONGA* continuous overlapping net glycemic action, *TIR* time in range, *MODD* mean of daily differences, *AGP* average glucose profile, *IQRs* interquartile ranges, *SMBG* self-monitoring of blood glucose, *CGM* continuous glucose monitoring

### Long‐term GV

Long-term GV is usually based on visit-to-visit measurements of HbA1c, FPG and PPG, with the subsequent calculation of their coefficient of variation (CV) and standard deviation (SD) [19]. Moreover, studies indicated that long-term GV was partly a reflection of surrounding hyperglycemia because measures of long-term GV correlated with either mean concentration of blood glucose or mean HbA1c [20, 21]. In recent years, variation independent of the mean (VIM), which was calculated based on logarithmic curve fitting (the natural logarithm of SD over the natural logarithm of the mean) to eliminate its correlation with mean level, was also used to measure long-term GV [22].

### Short‐term GV

Short-term GV characterized by sudden and rapid upward or downward glucose changes mainly includes within-day and between-day GV.

#### Within‐day GV

Similar to long-term GV, SD and CV are also the common metrics of short-term GV. When averaging daily SD or CV, the mean of within-day daily GV can also be estimated over the stated time [18]. Mean amplitude of glycemic excursions (MAGE) was the first to be developed, primarily to assess mealtime-related glucose excursions [23], and was the gold standard for assessing the short-term within-day GV [24]. Due to its simplicity, MAGE was widely used to assess within-day GV by measuring the arithmetic mean of the differences between consecutive peaks and nadirs. Mean absolute glucose (MAG) was another metric of within-day GV that summed absolute differences between sequential readings divided by the time between the first and last blood glucose measurement [8]. In addition, a novel measurement of within-day GV was presented by the continuous overlapping net glycemic action (CONGA) that calculated the SD of difference between a current blood glucose reading and a reading taken hours earlier [25]. Recently, time in range (TIR), referring to the percentage of time spent within the target glucose range of 3.9–10.0 mmol/L during a 24-h period, was identified as a key metric of within-day GV [26, 27].

#### Between‐day GV

Mean of daily differences (MODD) was considered to be the best metric for estimating the between-day GV [28]. This metric was based on the calculation of the absolute differences between two glucose values measured at the same time within a 24 h interval. Another metric of between-day GV was average glucose profile (AGP), which reflected the presence or absence of day-to-day synchrony in glucose patterns over a 14-day period and reported the results as interquartile ranges (IQRs) [29, 30].

Of note, particular attention should be given to the low blood glucose index (LBGI), high blood glucose index (HBGI) and average daily risk range (ADRR), as they were logarithmic calculations designed to evaluate the tendency to hypo or hyperglycemia, which reflected either within-day GV or between-day GV. LBGI and HBGI were preceded by a log transform to render symmetric the skewed distribution of glucose values to predict hypoglycemia or hyperglycemia [18, 31]. Meanwhile, ADRR was sum of the daily peak risks for hypo- and hyperglycemia [32].

Notably, the measuring method of GV was different in the last few years. The traditional approach to measuring GV relied on self-monitoring of blood glucose (SMBG) [20], but this method had been gradually replaced by continuous glucose monitoring (CGM) over the past few years [33–35]. Compared with SMBG, CGM with interstitial glucose measurements at 5 min intervals provides a more comprehensive record during the day and night periods [20, 34]. In recent years, Chico et al. reported that flash glucose monitoring, a new approach to glucose monitoring, had a long sensor lifetime of 14 days and emerged as a practical solution to the glucose monitoring [36].

## The role of GV in diabetic macrovascular and microvascular complications

There is growing evidence supporting that GV has drawn a great attention for its role in diabetic macrovascular and microvascular complications [15, 37–41]. Among type 2 diabetes mellitus (T2DM) patients from the Hoorn Diabetes Care System cohort, the individuals with a higher visit-to-visit GV had an unfavorable metabolic profile and had an increased risk of macrovascular and macrovascular complications as well as mortality [42].

### GV and diabetic macrovascular complications

It is generally accepted that macrovascular complications include coronary artery disease, peripheral vascular disease and cerebrovascular disease. A meta-analysis found that homeostasis model assessment of insulin resistance (HOMA-IR) and reduced intima-media thickness (IMT) level were the cardiovascular disease (CVD) risk factors and were significantly lower in low glucose variability group than in high glucose variability group [43]. Minimizing GV could improve insulin resistance and reduce IMT, consistent with a lowering in risk of CVD. Moreover, a post hoc cohort analysis including 160 patients with or without diabetes mellitus showed that post-procedural GV assessed by calculating the mean daily δ blood glucose during the first 2 days after transcatheter aortic valve implantation was associated with an increased risk of macrovascular complications (e.g., death, stroke and myocardial infarction) [44]. Similarly, a retrospective study enrolling 2215 patients who underwent coronary artery bypass grafting reported that increased 24-h but not 12-h postoperative GV was a predictor of major adverse events [45]. Benalia et al. revealed that T2DM patients admitted for acute myocardial infarction with elevated GV had significantly higher SYNTAX scores [46]. SYNTAX scores, which characterized coronary artery lesion severity, were independently related to high GV beyond HbA1c levels, suggesting that GV was associated with coronary artery disease severity and the early evaluation of GV might serve as a therapeutic target for both primary and secondary prevention [46]. Analogously, intraday GV calculated by MAGE was independently associated with coronary artery spasm in patients with dysglycemia [47]. Another meta-analysis showed that higher MAGE at admission was associated with higher risk of major adverse cardiovascular events in coronary artery disease patients regardless of the diabetic status [48]. Although GV was correlated with macrovascular complications to some extent, combining GV and HbA1c might exert incremental effects. Nusca et al. found that combining GV and HbA1c could identify the individuals at higher thrombotic risk among patients with T2DM undergoing percutaneous coronary intervention [49]. Recently, the Glycemia in Acute Stroke II (GLIAS-II) translational study was performed to evaluate the impact of GV on acute ischaemic stroke (IS) outcomes and examine the impact of intravenous or subcutaneous insulin on GV in an animal model of IS by using continuous subcutaneous monitoring devices [50], which might overcome the main limitations of the prior studies.

Consistent with the metrics of short-term GV, long-term GV was also correlated with the diabetic macrovascular complications. A prospective study by Gerbaud and his colleagues found that long-term GV assessed by SD during initial hospitalization was the strongest independent predictive factor for midterm major cardiovascular events in patients with diabetes [51]. Similarly, another prospective cohort study including 53,607 Chinese participants reported that elevated visit-to-visit FPG variability defined as the CV of FPG significantly increased the risk of CVD and all-cause mortality [9], which was helpful for predicting the risk for CVD and all-cause mortality. A prospective cohort study including 455 patients with T2DM and with follow-up for a median of 4.7 years identified that FPG variability calculated by CV could be a novel risk factor for the long-term adverse changes in left cardiac structure and systolic function in patients with T2DM [52]. Even after additionally stratified by mean HbA1c levels, FPG-CV was still independently associated with the annualized changes in left cardiac structure and systolic function in patients with HbA1c ≥ 7%, while not in patients with HbA1c < 7%. In the Veteran Affairs Diabetes Trial (VADT), FPG variability evaluated by CV and average real variability was significantly associated with CVD even after adjusting for the risk factors in patients with T2DM [10]. Moreover, Coronary Artery Risk Development in Young Adults (CARDIA) study suggested that higher long-term FPG variability assessed by CV during young adulthood before the onset of diabetes was associated with incident diabetes, macrovascular events and mortality [53]. Recently, Lee et al. showed that long-term FPG variability calculated by VIM was correlated with the risk of stroke, myocardial infarction, and all-cause mortality in patients with diabetes [54]. More importantly, the impact of FPG variability was higher in the elderly and those with a longer duration of diabetes and lower FPG levels. Yang et al. also found that visit-to-visit FPG variability measured by CV was an independent predictor of incidence of left ventricular adverse remodeling in T2DM patients with ST-segment elevation myocardial infarction [55]. Assessing FPG variability by other two measures, including SD and VIM, yielded similar findings. Along with the variability of FPG, long-term variability of HbA1c was also associated with the risk of macrovascular complications. A previous study enrolling 632 patients with T2DM revealed that long-term HbA1c variability (assessed by CV and VIM) and systolic blood pressure contributed to a combined and additive risk for CVD in patients with T2DM [56]. In Chinese T2DM patients, long-term HbA1c variability was calculated as intra-individual mean, SD, CV and adjusted SD, and was associated with macrovascular complication [57], and long-term stabilization of glucose is important in diabetes management, especially in the early stage of atherosclerosis. Meaningfully, a retrospective cohort study provided a support that HbA1c variability evaluated by SD and CV was associated with the presence of new-onset symptomatic heart failure with preserved ejection fraction (HFpEF) in patients with T2DM [58]. Analogously, Gu et al. showed that higher HbA1c variability (measured by SD and CV) was associated with higher all-cause mortality or composite endpoints, and was an independent predictor of all-cause mortality or composite endpoints [59]. Interestingly, Yokota et al. found the consistent results and suggested that reducing GV might represent a potential new therapeutic strategy for the prevention of HFpEF in T2DM patients [60]. Recent studies also addressed the importance of long-term HbA1c variability. The study enrolled 420 T2DM patients and suggested that visit-to-visit HbA1c variability expressed as SD, CV and VIM was independently associated with incidence of in-stent restenosis in patients with T2DM after stent implantation [61]. Of note, several studies disclosed that long-term GV including both HbA1c and FPG variability (calculated by CV) was associated with peripheral artery disease risk and accelerated progression of coronary atherosclerosis in patients with T2DM [62, 63]. These clinical results addressed the essential role of GV in diabetic macrovascular complications (Table 2), and paved the way for the research on relevant mechanisms.

**Table 2:** The role of GV in diabetic macrovascular

Metrics of GV	Measuring method	Individuals	Main results	References
Mean daily δ blood glucose	SMBG	160 patients with or without diabetes	Increased risk of macrovascular complications	[44]
MAGE	SMBG	204 patients with poorly controlled T2DM	Associated with coronary artery disease severity	[46]
MAGE	Flash glucose monitoring	50 patients with dysglycemia	Positively correlated with coronary artery spasm	[47]
MAGE and CV	CGM	35 T2DM patients on clopidogrel therapy	Provided additional diagnostic significance in identifying diabetic patients with HPR	[49]
SD of blood glucose	SMBG	327 patients with diabetes and acute coronary syndrome	An independent predictive factor for midterm major cardiovascular events	[51]
CV of FPG	SMBG	455 patients with T2DM	A novel risk factor for left cardiac structure and systolic function	[52]
CV and average real variability of FPG	SMBG	1791 individuals with T2DM	Significantly associated with cardiovascular disease	[10]
CV of FPG	SMBG	3769 individuals	Significantly associated incident diabetes, macrovascular events and mortality	[53]
VIM of FPG	SMBG	624,237 subjects with diabetes	Increased the risk of stroke, myocardial infarction, and all-cause mortality	[54]
CV of visit-to-visit FPG	SMBG	437 patients with T2DM and ST-segment elevation myocardial infarction	Independently predicted the incidence of left ventricular adverse remodeling	[55]
CV and VIM of HbA1c	SMBG	632 patients with T2DM and no history of cardiovascular disease	Increased the combined and additive risk for cardiovascular disease	[56]
Intra-individual mean, SD and CV of HbA1c	SMBG	5278 diabetic patients with no history of cardiovascular disease and atherosclerosis	Positively associated with macrovascular complications	[57]
SD and CV of HbA1c	SMBG	201 subjects with T2DM and arterial hypertension	Potentially predicted the progression of HFpEF	[58]
SD and CV of HbA1c	SMBG	902 patients with heart failure and T2DM	An independent predictive factor of all-cause mortality or composite endpoints	[59]
SD of HbA1c	CGM	100 type 2 diabetic patients with preserved left ventricular ejection fraction	Associated with poor left ventricular diastolic dysfunction	[60]
CV, SD and VIM of HbA1c	SMBG	420 diabetic patients after stent implantation	Independent predicted the incidence of in-stent restenosis	[61]
CV of HbA1c and FPG	SMBG	396 patients with T2DM	Positively associated with accelerated progression of coronary atherosclerosis	[62]
CV of HbA1c and FPG	SMBG	63,084 Chinese individuals with diabetes	Increased peripheral artery disease risk	[63]

*SMBG* self-monitoring of blood glucose, *MAGE* mean amplitude of glycemic excursions, *T2DM* type 2 diabetes mellitus, *CV* coefficient of variation, *CGM* continuous glucose monitoring, *HPR* high platelet reactivity, *SD* standard deviation, *FPG* fasting plasma glucose, *VIM* variation independent of the mean, *HFpEF* heart failure with preserved ejection fraction

### GV and diabetic microvascular complications

Diabetic nephropathy (DN), diabetic peripheral neuropathy (DPN) and diabetic retinopathy (DR) are the main microvascular complications caused by chronic hyperglycemia [6]. As with diabetic macrovascular complications, GV also played a crucial role in diabetic microvascular complications (Table 3).

**Table 3:** The role of GV in diabetic microvascular

Metrics of GV	Measuring method	Individuals	Main effects	References
SD of HbA1c	SMBG	4231 patients with T2DM and albuminuria	Increased the risk of albuminuria	[65]
CV of HbA1c	SMBG	1383 T2DM patients	An independent risk factor for deterioration of renal function	[66]
SD of HbA1c	SMBG	388 patients with diabetes and chronic kidney disease	Positively associated with the risk of chronic kidney disease progression	[68]
SD of HbA1c	SMBG	604 patients with T2DM	Significantly associated with progression of DN	[69]
MAGE	CGM	40 patients with T1DM or T2DM	An independent risk factor for DPN	[70]
TIR	Flash glucose monitoring	364 individuals with diabetic peripheral neuropathy	Negatively correlated with the risk of painful DN	[71]
CV of visit-to visit FPG	SMBG	2773 patients with T2DM	Increased the risk of DPN	[72]
CV of visit-to visit FPG	SMBG	36,152 individuals with T2DM	Potent predictors of DPN	[73]
CV and mean of HbA1c	SMBG	563 T2DM patients	Significantly increased the risk of DPN	[74]
Intrapersonal mean, SD and CV of HbA1c	SMBG	238 patients with T2DM	Strongly associated with the degree of severity of cardiovascular autonomic neuropathy	[75]
Intrapersonal mean, SD and CV of HbA1c	SMBG	223 patients with T2DM	Strongly associated with the severity of peripheral neuropathy	[76]
TIR	CGM	3262 patients with T2DM	Inversely correlated with the severity of DR	[78]
CV and SD of HbA1c	SMBG	220 patients with T1DM	Positively associated with DR and impaired renal function	[80]
CV of HbA1c	SMBG	415 patients with T1DM	Independently associated with the risk of DR development	[81]

*SD* standard deviation, *SMBG* self-monitoring of blood glucose, *T2DM* type 2 diabetes mellitus, *CV* coefficient of variation, *DN* diabetic neuropathy, *MAGE* mean amplitude of glycemic excursions, *CGM* continuous glucose monitoring, *DPN* diabetes peripheral neuropathy, *T1DM* type 1 diabetes mellitus, *TIR* time in range, *FPG* fasting plasma glucose, *DR* diabetic retinopathy

#### The role of GV in DN

An analysis based on three large and well-designed clinical trials demonstrated a consistent finding that FPG variability was correlated with increased risk for moderate to severe DN [64]. Within the Association of Clinical Diabetologists Annals database, Ceriello et al. identified that high variability in HbA1c (assessed by SD) conferred the highest risk of developing albuminuria, contributing to the development of diabetic kidney disease [65]. Similarly, another study also confirmed that HbA1c_CV was an independent risk factor for deterioration of renal function, and early minimization of GV could curb deterioration of renal function [66]. Subsequent studies addressed that the long-term intra-individual variability in HbA1c, lipid parameters, uric acid and blood pressure played a greater role in the progression of chronic kidney disease (CKD) than the absolute value of each single variable, clarifying the important role of long-term intra-individual variability in progression of CKD [67]. A longitudinal study showed that greater HbA1c variability with a decreasing trend of HbA1c was defined as the SD of HbA1c and was associated with a lower risk of progression to dialysis in the patients with stages 3–4 CKD and poor glycemic control [68]. Noteworthily, there were differences in the risk factors for the progression of DR and DN in T2DM, and an observational study discovered that average HbA1c was significantly associated with progression of DR, whereas HbA1c variability (evaluated by SD) was significantly associated with progression of DN [69]. However, the initiation and progression of albuminuria are not included in the definition of DN progression in this observational study. Strikingly, Lachin et al. showed that within-day GV, as determined from quarterly glucose profiles, did not participant in the development of microvascular complications [20]. Thus, further prospective studies are required to confirm these discordances.

#### The role of GV in DPN

Short-term GV was estimated by MAGE in CGM and was found to be independently associated with a higher risk of DPN with type 1 or 2 diabetes [70], but the study had a small sample size, which might not be able to evaluate patients with severe diabetes complications. Yang et al. found that a decreasing level of TIR was significantly associated with an increasing risk of painful diabetic neuropathy, which might be underscored as a valuable clinical evaluation measure [71]. Investigators of a retrospective study reported that long-term variability as evaluated by FPG-CV was associated to the risk of painful DPN in patients with T2DM [72]. Consistent with this result, several studies found that HbA1c, FPG-CV and HbA1c-CV increased risks of DPN and were potent predictors of DPN in T2DM patients [73, 74], which might play a crucial role in clinical risk assessments. Recent studies revealed that HbA1c variability calculated by SD was independently associated with the severity of peripheral neuropathy and cardiovascular autonomic neuropathy in patients with T2DM [75, 76]. Conversely, a cross-sectional study including 133 young adults with type 1 diabetes mellitus (T1DM) suggested that GV might not be a risk factor for diabetic neuropathy [77]. Longitudinal studies are required to confirm the elaborated role of GV in the progression of DPN.

#### Roles of GV in DR

Among a total of 3262 patients with T2DM, Lu et al. indicated that TIR measured by CGM was significantly associated with all stages of DR [78]. In the Rio De Janeiro Type 2 Diabetes Cohort Study, long-term visit-to-visit GV, particularly the 24-month parameters either estimated by HbA1c or FPG, could predict retinopathy progression in patients with good glycemic control (HbA1c ≤ 7.5%, 58 mmol/mol) and predicted new-incident peripheral neuropathy [38]. A recent meta-analysis showed that high FPG variability (assessed by median or mean FPG variability levels) was strongly associated with the risk of retinopathy [odds ratio (OR) = 3.68; 95% CI 1.01–13.4] in patients with T2DM [79]. Nevertheless, for elderly patients with T2DM, FPG variability did not increase the progression of DR [79]. On the other hand, long-term variability of HbA1c assessed by CV or mean value was closely associated with DR (OR: 8.93; 95%CI 1.86–42.87), suggesting that both good and stable glycemic status might be important to prevent microvascular complications [80]. Due to the wide confidence intervals and the high heterogeneity, further studies are needed to confirm these conclusions. Recently, Schreur et al. performed a long duration of follow-up study and found that long-term HbA1c variability (defined as CV) was one of the risk factors for the development and progression of DR in patients with T1DM [81].

## Relevant mechanisms of GV in diabetic macrovascular and microvascular complications

Although accumulated clinical evidence described the association of GV and diabetic macrovascular and microvascular complications, the relevant mechanisms are multiple and indistinct. Previous studies demonstrated that GV was associated with the risk of both hyperglycemia and hypoglycemia [32, 82–84]. Increasing evidence has shown that GV, hypoglycemia and hyperglycemia are all closely related to oxidative stress [85, 86]. It is noteworthy that transient hyperglycemia has been shown to induce even more vascular damage than sustained hyperglycemia, mainly mediated by oxidative stress [87, 88]. Further, several researches indicated that transient hyperglycemia might cause epigenetics changes, such as cellular metabolic memory [89, 90], increasing insulin resistance and pancreatic β-cell dysfunction and apoptosis [91, 92]. Strikingly, Costantino et al. demonstrated that MAGE was independently associated with adverse epigenetic signatures on p66^Shc^ promoter and promoted chromatin changes, leading to persistent vascular dysfunction in patients with T2DM and with target HbA1c levels [93]. Intriguingly, an animal experiment also demonstrated that higher GV displayed a more pronounced reactive oxygen species production and endothelial dysfunction [94]. More importantly, short-term glycemia fluctuations were reported to induce superoxide overproduction, inflammatory cytokines generation, increased oxidative stress and endothelial dysfunction and damage [87, 95, 96], which contributed to chronic diabetic complications. Although oxidative stress has been considered as one of the underlying mechanisms for the effects of GV on diabetic complications [96–98], several studies have shown conflicting results [99, 100]. These inconsistent results may be attributed to the differences in medications and the dissimilar methods used to determine oxidative stress, and further prospective researches are warranted to figure out these inconsistencies.

**Fig. 1:**
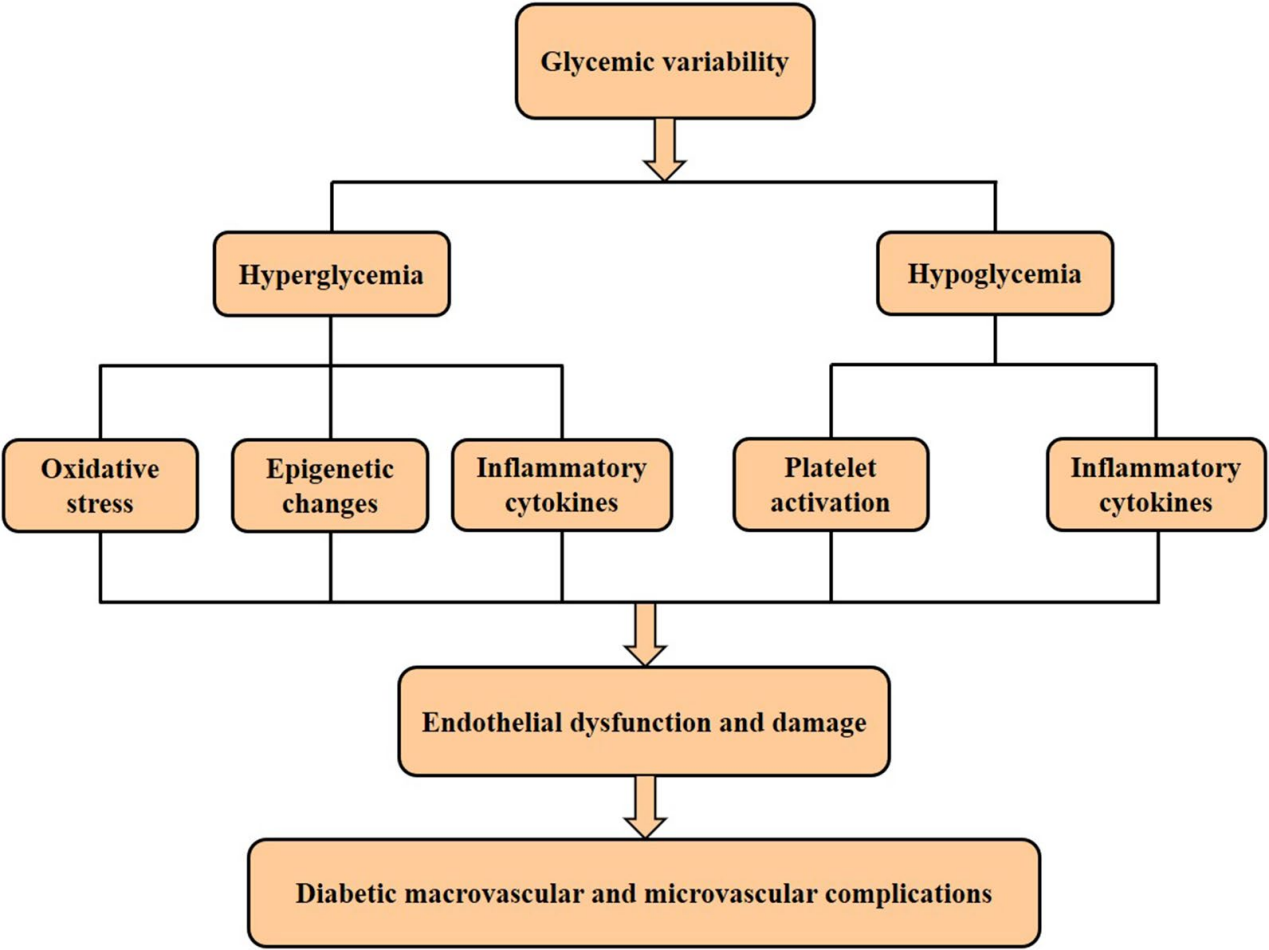
Potential mechanisms of glycemic variability in diabetic macrovascular and microvascular

High GV has also been proven to be associated with the risk of hypoglycemia, which might be an independent cause of cardiovascular damage. Potential mechanisms by which hypoglycemia could lead to an increase in cardiovascular risk were manifested by release of inflammatory cytokines, increased platelet activation and endothelial dysfunction [101, 102]. Collectively, these results suggest that high GV increases the risk of hyperglycemia and hypoglycemia, subsequently inducing oxidative stress, inflammatory cytokines generation, epigenetics changes and endothelial dysfunction and damage, ultimately contributing to diabetic complications (Fig. 1).

## Mechanism‐based therapeutic strategies

There are several possible mechanism-based non-pharmacological and pharmacological strategies for reducing GV in clinical practice (Table 4).

**Table 4:** Mechanism-based therapeutic strategies for reducing GV

Strategies	Population	Possible mechanisms	References
Non-pharmacological strategies			
CGM	40 patients with T1DM	Minimized the risk of severe hypoglycemia	[104]
High-intensity interval training and moderate-intensity continuous training	15 inactive overweight or obese women	Decreased endothelial cell damage	[108]
Aerobic and eccentric exercise	16 healthy subjects	Reduced inflammatory cytokines and oxidative stress markers	[109]
Low carbohydrate diet	10 patients with T1DM	Resulted in more time in euglycemia, less time in hypoglycemia	[110]
Pharmacological strategies			
Once-weekly trelagliptin and once-daily alogliptin	27 patients with T2DM	Improved glycemic control and reduced GV without inducing hypoglycemia	[114]
GLP-1 RA with basal insulin	160 patients with T2DM	Lowered hypoglycemia and might contribute to the cardiovascular outcome reduction	[115]
DPP4 inhibitors combined with metformin	69 patients with T2DM	Reduced GV and hypoglycemia	[116]
DPP4 inhibitors combined with metformin	34 patients with T2DM	Reduced GV and hypoglycemia	[118]
Metformin plus vildagliptin	44 patients withT2DM	Attenuated oxidative stress index	[119]
Empagliflozin as adjunct to insulin	75 patients with T1DM	Decreased glucose exposure and variability and increased time in glucose target range.	[120]
SGLT2 inhibitors	15 patients with T1DM	Improved TIR and the mean glucose level and SD	[121]

*CGM* continuous glucose monitoring, *T1DM* type 1 diabetes mellitus, *T2DM* type 2 diabetes mellitus, *GV* glycemic variability, *GLP-1 RA* glucagon-like peptide 1 receptor agonist, *DPP4* dipeptidyl-peptidase 4, *SGLT2* sodium–glucose cotransporter 2

### Non‐pharmacological strategies

CGM, either from real-time use or intermittently viewed, has beneficial effects on metabolic control, reducing risks of hyperglycemia and hypoglycemia, and decreasing GV, mean glucose concentration, and HbA1c values [103, 104]. The international consensus on the use of CGM highlighted the importance of assessing and reporting the percentages of TIR, time above range (TAR) and time below range (TBR) in conjunction with the evaluation of glucose control [34]. Moreover, a recent meta-analysis found that CGM could improve glycemic control by expanding TIR and decreasing GV, TBR and TAR in diabetes [105]. Additionally, previous studies suggested that exercise training, including resistance exercise and aerobic exercise, reduced GV and oxidative stress levels in patients with T2DM [106, 107]. Analogously, a recent study disclosed that two weeks of both high-intensity interval training and moderate-intensity continuous training decreased GV and endothelial cell damage in obese women at elevated risk of T2DM [108]. Of note, both aerobic and eccentric exercise reduced GV in healthy individuals, which might be mediated by inflammatory cytokines and stress oxidative markers [109]. Another non-pharmacological strategy is dietary interventions. Low carbohydrate diet appeared to be sufficient to reduce postprandial hyperglycemia and improve glucose fluctuation, resulting in more time in euglycemia, less time in hypoglycemia and less GV [110–113].

### Pharmacological strategies

Glucose-lowering drugs that achieve a target HbA1c and decrease the risk of hypoglycemia are crucial for the management of diabetes. A randomized pilot study concluded that once-weekly trelagliptin and once-daily alogliptin reduced GV and improved glycemic control without inducing severe treatment-emergent adverse events and hypoglycemia [114]. Particularly, greater benefits are shown in therapies combing new glucose-lowering drugs with metformin or insulin. The combination of basal insulin with a glucagon-like peptide 1 receptor agonist (GLP-1 RA) displayed the lowest GV and hypoglycemia in patients with T2DM, which might contribute to a reduction of cardiovascular outcome [115]. Furthermore, dipeptidyl peptidase 4 (DPP4) inhibitors combined with metformin therapy improved glucose level with a significantly greater reduction in GV and hypoglycemia [116–118]. Subsequently, a current study concluded that metformin plus vildagliptin therapy was more effective than metformin monotherapy by attenuating oxidative stress index [119]. Consistent results were obtained when combined sodium glucose cotransporter 2 (SGLT2) inhibitors with insulin therapy [120, 121]. Empagliflozin as adjunct to insulin decreased glucose exposure and variability, as well as increased time in glucose target range in patients with T1DM [120]. Moreover, a retrospective cohort study unraveled that SGLT2 inhibitors improved TIR, SD and the mean glucose level without increasing the TBR < 70 mg/dL in patients with T1DM [121]. In short, new antidiabetic drugs combined with basal insulin or metformin might be preferred pharmacological strategies for reducing hypoglycemia and oxidative stress, thus decreasing the incidence of diabetic complications.

## Conclusions

With the improved availability of new glucose monitoring technologies, such as CGM and flash glucose monitoring, GV is becoming a more meaningful metric of glycemic control, and is without doubt now being recognized. Elaborating the role and mechanisms of GV in diabetic macrovascular and microvascular complications will be conducive to taking targeted measures in clinical practice and providing the crucial help for clinicians to manage the diabetes-related complications.

## Data Availability

Not applicable.

## Abbreviations

GV: Glycemic variability
FPG: Fasting plasma glucose
PPG: Postprandial glucose
CV: Coefficient of variation
SD: Standard deviation
VIM: Variation independent of the mean
MAGE: Mean amplitude of glycemic excursions
MAG: Mean absolute glucose
CONGA: Continuous overlapping net glycemic action
TIR: Time in range
MODD: Mean of daily differences
AGP: Average glucose profile
IQRs: Interquartile ranges
LBGI: Low blood glucose index
HBGI: High blood glucose index
ADRR: Average daily risk range
SMBG: Self-monitoring of blood glucose
CGM: Continuous glucose monitoring
T2DM: Type 2 diabetes mellitus
HOMA-IR: Homeostasis model assessment of insulin resistance
IMT: Intima-media thickness
CVD: Cardiovascular disease
GLIAS-II: Glycemia in Acute Stroke II
IS: Ischaemic stroke
VADT: Veteran Affairs Diabetes Trial
CARDIA: Coronary Artery Risk Development in Young Adults
HFpEF: Heart failure with preserved ejection fraction
DN: Diabetic nephropathy
DPN: Diabetes peripheral neuropathy
DR: Diabetic retinopathy
CKD: Chronic kidney disease
T1DM: Type 1 diabetes mellitus
TAR: Time above range
TBR: Time below range
GLP-1 RA: Glucagon-like peptide 1 receptor agonist
DPP4: Dipeptidyl-peptidase 4
SGLT2: Sodium glucose cotransporter 2
